# Wideband and Wide Beam Polyvinylidene Difluoride (PVDF) Acoustic Transducer for Broadband Underwater Communications †

**DOI:** 10.3390/s19183991

**Published:** 2019-09-16

**Authors:** Marcos S. Martins, Carlos L. Faria, Tiago Matos, Luís M. Goncalves, José Cabral, António Silva, Sérgio M. Jesus

**Affiliations:** 1 MEMS-UMinho, University of Minho, Campus of Azurém, 4800-058 Guimarães, Portugal; carlosfaria@dei.uminho.pt (C.L.F.); b7567@dei.uminho.pt (T.M.); lgoncalves@dei.uminho.pt (L.M.G.); cabral@dei.uminho.pt (J.C.); 2 LARSyS, University of Algarve Campus de Gambelas, 8005-139 Faro, Portugal; asilva@ualg.pt (A.S.); sjesus@ualg.pt (S.M.J.)

**Keywords:** polymer ultrasound transducer, PVDF acoustic emitter, wideband acoustic emitter, wide beam acoustic emitter, very high frequency acoustic emitter, acoustic broadband communications, underwater wireless communications

## Abstract

The advances in wireless communications are still very limited when intended to be used on Underwater Communication Systems mainly due to the adverse proprieties of the submarine channel to the acoustic and radio frequency (RF) waves propagation. This work describes the development and characterization of a polyvinylidene difluoride ultrasound transducer to be used as an emitter in underwater wireless communications. The transducer has a beam up to 10° × 70° degrees and a usable frequency band up to 1 MHz. The transducer was designed using Finite Elements Methods and compared with real measurements. Pool trials show a transmitting voltage response (TVR) of approximately 150 dB re µPa/V@1 m from 750 kHz to 1 MHz. Sea trials were carried in Ria Formosa, Faro (Portugal) over a 15 m source—receiver communication link. All the signals were successfully detected by cross-correlation using 10 chirp signals between 10 to 900 kHz.

## 1. Introduction

Acoustic communication systems have been widely used in underwater environments, since acoustic waves have low attenuation at low frequencies (up to tens of kHz), reaching large distances. Acoustic waves propagate more easily in an underwater environment [1] than the radio frequency (RF) and optic waves, but attenuation, ambient noise, Doppler Effect, low and variable sound speed, multipath and sound refraction (scattering) air bubbles and particles in suspension represents a considerable obstacle in underwater wireless communications [2]. One solution for higher data rates is to increase the carrier frequency [3]. However, increasing the frequency also will increase the attenuation and this represents a major drawback. To give some perspective, an acoustic signal at 1 MHz is attenuated around 280 dB/km considering only the attenuation by absorption [4].

Despite the acoustic communication advantages in an underwater environment, there is no reliable solution for broadband wireless communications underwater. There are some works with RF and optics for underwater short-range and high data rate communications, but RF is highly attenuated due to conductivity proprieties of water [5] and optics rely on transparent and clear water to propagate [6]. Therefore, there is a technological gap concerning high data rate wireless communications for subaquatic applications. In the sense of developing communication systems using transducers with characteristics of a wideband, wide beam, and high frequency; there are several works in the literature that address this issue. 

For example, a wide-band transducer was presented by Minoru Toda [7] consisting of a polyvinylidene difluoride (PVDF) coiled film attached to a disc. The film is excited with an electric potential along the thickness but the displacement is affected along the coil, which results in the disc vibration. However, the transducer only has a broadband response for frequencies below 100 kHz and does not allow the control of the beam angle. 

Another work is presented by S. Zhang [8] and reported the development of a piston transducer with two resonance points between 90 and 220 kHz, referred as the Transverse Resonance Orthogonal Beam (TROB) mode, where the active material is set in resonance in half-wavelength mode in the transverse direction and the acoustic beam is generated in the conventional transverse width beam direction and latter being orthogonal to the resonating transverse direction.

A more recent work presented by S. Hao [9] describes the development of a broadband and omnidirectional emitter transducer. A cylindrical transducer was developed by using piezoelectric ceramic elements alternating with a flexible polymer and a matching layer for multimode coupling. After testing, the working frequency range of the transducer was between 230–380 kHz.

Despite the recent developments presented in the literature about acoustic transducers, there is no reliable solution that meets all the needs in underwater broadband wireless communications for a distance coverage range up to 15 m [10]. In this sense, and in order to fill this technological gap, the present work describes the development and implementation of a wideband wide beam PVDF acoustic transducer to operate as emitter for frequencies up to 1 MHz. So, this transducer will be a key element in a high data rate wireless communications system for short distances (up to 15 m) [11]. 

The paper is organized as follows: Section 2 summarizes the basic concepts of piezoelectric acoustic transducers. Section 3 describes the material selection and the transducer design. Section 4 presents the transducer simulation using finite elements and Section 5 describes the fabrications process and electrical and acoustical characterization. Section 6 describes the experimental setup and field test results. Finally, in Section 7, some conclusions are drawn. 

This article is an extended version of a preliminary work published in [12]. We extend our previous work by increasing the introduction and transducer design background detail, include an extended analysis of the transducer FEM simulation with individual results for 250 kHz, 500 kHz, 750 kHz and 1 MHz frequencies. Detailed information about the acoustic characterization experimental setup and we include an electric characterization. A Sea trial for real-world performance test was carried out in a 15 m communication link in Ria de Formosa, Faro—Portugal. 

## 2. Piezoelectric Transducers Design Background

In order to fulfill all the proposed objectives, it is necessary to understand a set of operational characteristics of piezoelectric materials and acoustic transducers. Conventional transducer design is normally dominant in one key characteristic: omnidirectional or high frequency, but when combining wideband, high frequency and wide beam, it will require a well-balanced compromise between all characteristics.

When designing an ultrasonic transducer, one of the first steps is to define the resonance frequency and the Q factor. The Q factor is obtained by dividing the resonance frequency by the bandwidth (Hz). The resonance effect in an acoustic transducer is obtained by the mismatch of acoustic impedance between the transducer and the medium which results in an internal reflection of acoustic energy inside the transducer. The transducer reflection coefficient is the quantity of acoustic energy (in percentage) retained inside the transducer [13] and can be obtained by:

(1) $$R={\left(\frac{Z_{2}-Z_{1}}{Z_{2}+Z_{1}}\right)}^{2}$$

where *Z*_1_ is the medium acoustic impedance and *Z*_2_ is the transducer acoustic impedance. When the piezoelectric drive signal and the reflected energy are synchronized, the resonance effect is achieved. So, for a wideband transducer, a low Q factor is desirable and for that, it is better to avoid the resonance effect by matching the acoustic impedance of the transducer and the medium. The acoustic impedance of a material is defined as the product of its density and acoustic velocity. 

For designing a wide beam transducer, it is necessary to consider the piezoelectric material composition and size since they will influence the beam divergence angle *δ* [13], which can be obtained by:

(2) $$\delta =arcsin(\frac{\lambda }{D})$$

where *D* is the transducer diameter when considering a cylindrical transducer (in a more general context it should be considered the length exposed the water) and *λ* the wavelength. Considering Equation (2), in high frequency transducer with wide beam pattern, the resulting diameter is minuscule compared to the transducer surface area needed to achieve 15 m distance range since the transducer acoustic power (P) is directly proportional to the surface area of the piezoelectric element *A_P_*.

(3) $$P=A_{P}I$$

where *I* is the intensity of the acoustic pressure wave and can be obtained by:

(4) $$I=\frac{p^{2}}{c\rho }$$

where *p* is the pressure wave, *c* is the sound speed and *ρ* is the propagation medium density both assumed constant in space and time. 

Usually, at high frequencies, piezoelectric ultrasonic transducers operate in the thickness mode, which means that the deformation is along the polarization axis and the excitation electric field is in the same direction. The free displacement of the material (*ξ*), without restraining force and assuming uniform strain over the surface [14], is given by:

(5) $$\xi =d_{33}vn$$

where *v* is the applied voltage, *n* is the number of layers and *d*_33_ is the coupling coefficient in the thickness direction. The deformation creates a pressure wave in the medium [13], whose force amplitude F can be obtained by:

(6) $$F=A_{p}p$$

where *p* is the pressure wave and can be obtained by:

(7) $$p=\sqrt{2\;}\left(\pi c\rho f\xi \right)$$

where *ρ* is the piezoelectric material density and *f* is the sound wave frequency. 

The maximum force that the piezoelectric element can apply to the medium is obtained by:

(8) $$F=d_{33}\frac{A_{p}}{S_{33}^{E}t_{p}}v$$

where $S_{33}^{E}$ is the elastic compliance coefficient and *t_p_* is the thickness of a single layer [15].

To ensure optimal operation, the force that the transducer can apply to the medium must be higher than the resulting acoustic wave force generated by the transducer deformation, otherwise, the piezoelectric material will not be able to produce a homogeneous displacement across the entire surface, generating acoustic waves with low amplitude and distortion. Through Equations (6)–(8), it is possible to obtain the maximum stack thickness [3]:

(9) $$nt_{p}\leq \frac{1}{\sqrt{2}\pi c\rho S_{33}^{E}f}$$

This condition allows the calculation of the layer thickness where *n* is the number of layers for a specific frequency and material.

In conclusion, to fulfill the objectives of a wideband in a MHz frequency range with a wide beam pattern transducer, a piezoelectric material with a low acoustic impedance is necessary, a high coupling coefficient in a multilayer structure with a considerable surface area and a geometric shape that promotes the divergence of the acoustic beam. 

## 3. Material Selection and Transducer Design

Due to the good response at high frequencies, piezoelectric materials are frequently used in the ultrasound transducers assembly. The most common are the lead zirconate titanate (PZT), lead titanate (PT), lead magnesium niobate (PMN) and lead zinc niobate (PZN) in the ceramics family [16] and polyvinylidene difluoride (PVDF) and poly(vinylidene fluoride-co-trifluoroethylene) (P(VDF-TrFE)) in the polymers family [17,18]. There are the single crystals of PZT, PMN, and PZN that have been used [19]. 

The polymeric based solutions have the lowest acoustic impedance among all materials used in underwater acoustic transducers. One of the major advantages of using low acoustic impedance is related to the high transfer of energy between the transducer and the medium, decreasing the resonance effect significantly. The water’s acoustic impedance is around 10^6^ kg/m^2^s, the PVDF is 3.3 × 10^6^ kg/m^2^s and the PZT is around 31.5 × 10^6^ kg/m^2^s, which results in an internal reflection coefficient of 88% for PZT and around 28.7 % for PVDF [3], as presented in the Table 1. Table 1 allows for a comparative view of some characteristics of PZT-5H and PVDF which will be used in the simulations of Section 4.

The resonance effect reduction has two major consequences: it reduces the sound pressure output at the resonance point and increases the transducer usable bandwidth which is desirable for broadband digital communications [3]. Another important aspect of polymeric based solutions is the fact that PVDF is lead-free, which represents an advantage in terms of environmental impact. Considering wideband requirements, PVDF has been selected as the transducer active element.

**Figure 1:**
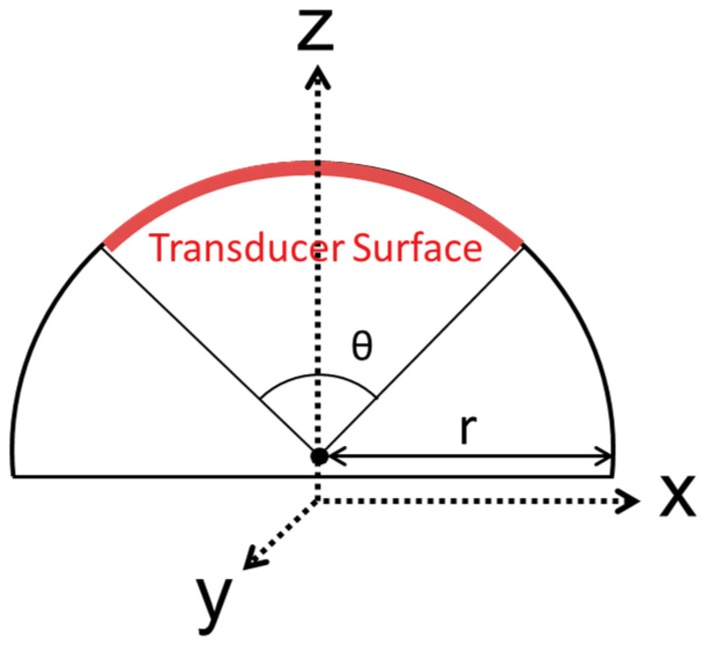
Half cylinder shape transducer 2D Model with the necessary piezoelectric active surface area (in red) to achieve a beam spread angle of θ [12].

PVDF has a low piezoelectric coefficient, almost 20 times lower than common piezo ceramics [4]. Nevertheless, it is possible to overcome this limitation by suitable transducer design using a laminated transducer structure, stacking several layers of PVDF films, it is possible to significantly increase the transducer performance [4]. Another possible solution is to increase the transducer surface area, but for the piston transducer, this will reduce the beam divergence angle, according to Equation (2). Therefore, it is necessary to implement a geometry that allows the transducer surface area to increase and also the beam divergence angle. According to Sherman [13], using curved geometries, it is possible to control the beam divergence angle and increase the surface area, where the area is practically unlimited, once the transducer surface area is proportional to the circumference radius, as shown in Figure 1.

Figure 1 shows a top view of a half-cylinder-shaped transducer with radius r, where the transducer length (in red) is equal to the arc length in the circular sector defined by the central angle θ.

The prototype dimensions were calculated taking into account the requirements in Table 2.

Taking into account Equation (9), for a 1MHz PVDF transducer, the maximum thickness is limited at 229 µm [4]. Therefore, the selected active element has two layers of 110 µm PVDF with silver electrodes [20]. According to the design in Figure 1, for a transducer to cover an area of 70° in XY plane and 10° in YZ plane, when using a cylinder with 7.5 cm radius, the active element has to be 1.7 cm wide and 9.2 cm long which correspond to 70° of the 47 cm circumference perimeters, according to Equation (2).

## 4. Finite Element Method Simulation

**Figure 2:**
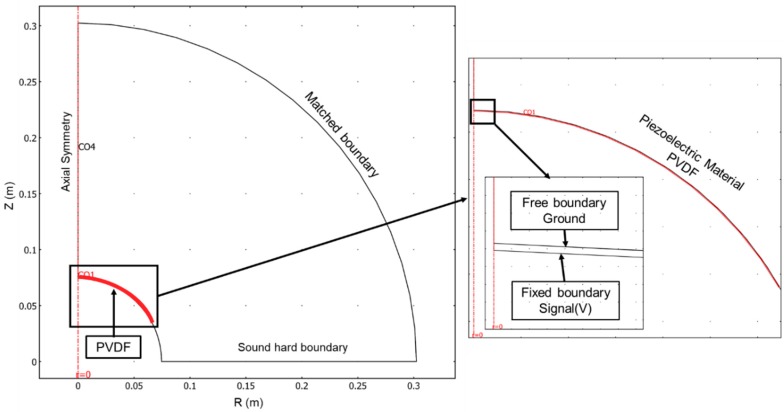
Boundary conditions definition for the 2D symmetric geometry FEM simulation, composed of a 30 cm radius medium and the transducer active element.

Before implementation, the transducer design was subject to a Finite Element Method (FEM) simulation, in order to estimate the geometry performance. The design model prototype was implemented in a COMSOL Multiphysics [16] platform in a 2D symmetric plane with the models Piezo Strain Plane for the active element actuation and the model Pressure Acoustic for the pressure waves. The selected mesh has particles with a triangular shape and with 300 µm size for the propagation medium and 200 µm size for the PVDF film. The simulation model consists of a 2D symmetrical slice of 30 cm radius environment, as presented in Figure 2. For accurate measurement comparison between the simulations and the real test, the ideal radius simulation should have 100 cm. However, the FEM simulation was very demanding in terms of processing power and memory, forcing a model to simulate only 30 cm. This limitation will prevent direct comparison between the FEM simulation and the experimental tests since the Transmission Voltage Response (TVR) standard measurements are performed at 1 m distance.

Figure 2 shows the boundaries defined in the symmetrical 2D slice model: Z has a symmetrical axial; R has a sound hard boundary (wall) and the curved outside surface has matched boundary to observe all acoustic energy (no reflections). On the right side of Figure 2, a zoom from the transducer piezoelectric film is presented, where it was defined for the upper boundary a free mechanic boundary electrically connected to the ground and for the lower boundary a fixed mechanic boundary electrically connected to the drive signal. The simulations were performed with the configurations of Table 3.

**Figure 3:**
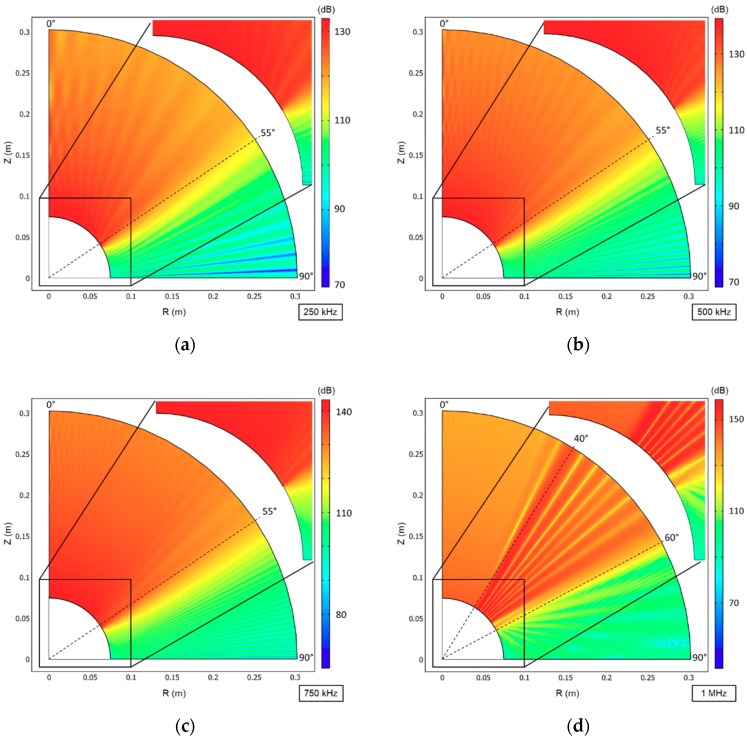
Sound Pressure Level (dB re 1 μPa) simulation results in a symmetrical axis for: (**a**) 250 kHz; (**b**) 500 kHz; (**c**) 750 kHz; (**d**) 1 MHz. The max responsive axis with a maximum deviation of 6 dB is marked by the dashed line.

Figure 3 shows the simulation results in a symmetrical half-plane which represents half transducer. The Z-axis was defined as the 0° axis and R axis the 90°. In terms of the beam spread angle, a maximum deviation of 6 dB was assumed with respect to the maximum peak of the Sound Pressure Level (SPL) (dB re 1 μPa).

The results show that the transducer exceeded the expected 70° for all frequencies, the beam spread angle (θ) reaches 110° for a maximum deviation of 6 dB. In the simulations, the SPL reaches a maximum of 134 dB, 141 dB and 143,5 dB for 250 kHz, 500 kHz and 750 kHz respectively, in the central lobe at 0°, and the loss of 6dB is only reached at 55°. In the 1 MHz simulation the main lobe is shifted to the right at 40° with 150 dB and the loss of 6dB is reached at 60°. In conclusion, simulation results are as expected, making this transducer design suitable for implementing a non-directional large beam transducer.

## 5. Implementation and Characterization

**Figure 4:**
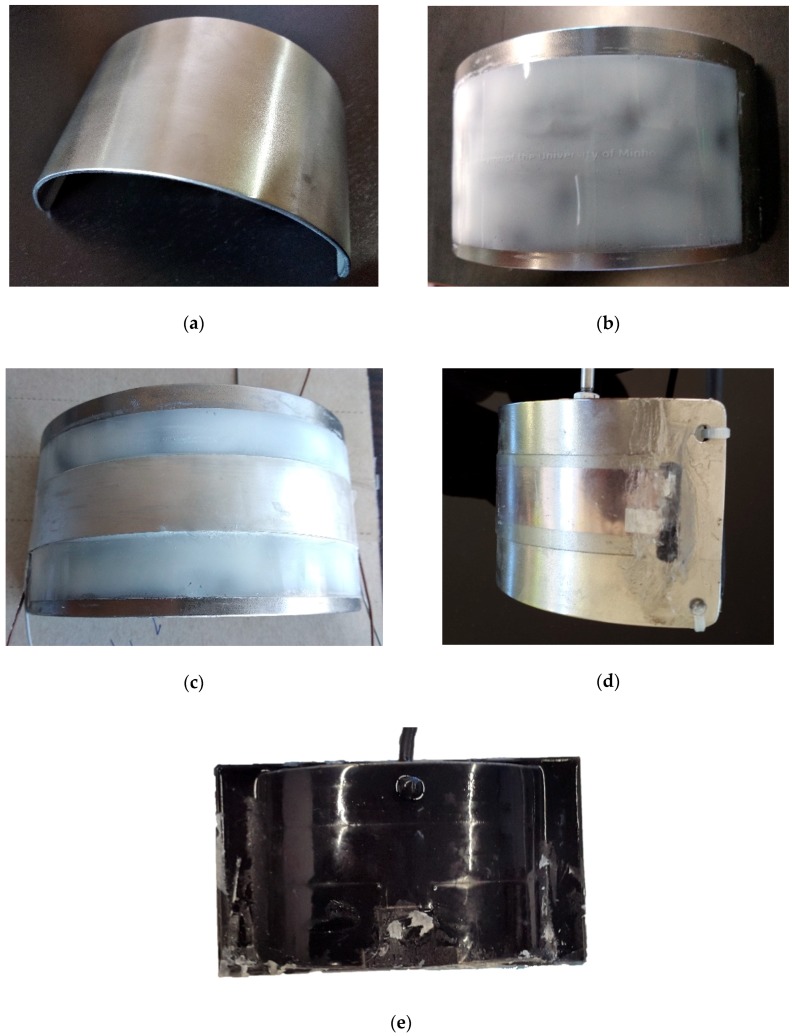
Photographic history of the transducer manufacturing: (**a**) curved stainless-steel sheet backing layer; (**b**) isolator layer, (**c**) polymer ultrasound transducer (PVDF) film; (**d**) waterproofing silicone layer and (**e**) final coat of black Polyurethane resin.

The transducer was implemented according to the dimensions and characteristics obtained in the simulation. Figure 4 shows the transducer construction progress.

A backing layer, composed by a curved stainless-steel sheet with 2 mm thickness, was prepared as shown in Figure 4a. The backing layer has the main function of projecting all acoustical energy in the desired direction, but it serves also in this particular case to fix the curved shape of the transducer. In the outer surface, an unpolarized PVDF isolator was glued to prevent an electric short-cut between the backing layer and the transducers electrodes, as shown in Figure 4b, then the PVDF films were glued using a thin layer of silicone in a curved shape frame, as showed in Figure 4c. The electrodes were connected to the conductive wires using aluminum tape and silver ink. For waterproofing, the PVDF films were covered with a thick layer of silicone, as shown in Figure 4d and finally the complete setup was covered with a coat of black Polyurethane resin type UR5041 as shown in Figure 4e.

### Electric and Acoustic Characterization

**Figure 5:**
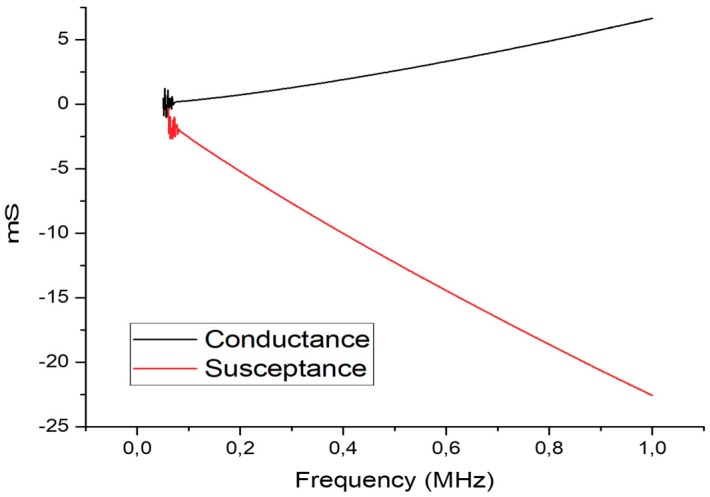
Admittance (conductance + susceptance) measurements from 60 kHz to 1 MHz using a network analyzer Keisight E5071C.

The electric characterization was performed using a network analyzer Keisight E5071C. Figure 5 shows the admittance graphic (conductance + susceptance). Measurements were performed from 60 kHz to 1 MHz with a step of 1 kHz.

Analyzing Figure 5, it is evident that the transducer has a strong capacitive component, due to the negative nature of the susceptance which represents the imaginary part of the admittance. Such capacitive component increases with frequency. This behavior was expected since the transducer is composed of two electrodes with an insulator PVDF core. Nevertheless, admittance has relatively low values (max 24 mS) which means the transducer will have a low power consumption.

**Figure 6:**
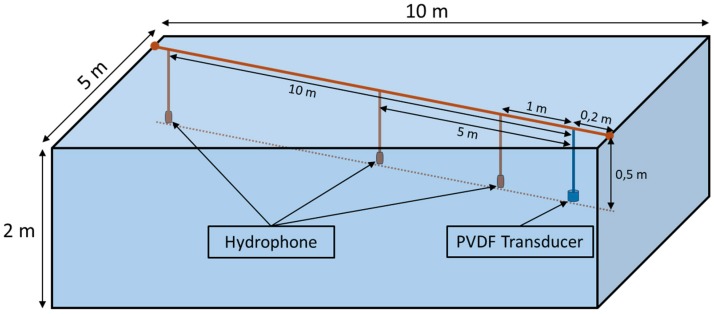
Experimental setup diagram used for measuring the Transmission Voltage Response and the Spread Angle Response. The transducer was tested in a freshwater pool with 10 m long, 5 m wide and 2 m deep with an average temperature of 21 °C.

For acoustic characterization, the transducer was tested as an emitter in a freshwater pool with 10 m long, 5 m wide and 2 m deep with an average temperature of 21 °C, using hydrophone as a receiver. To avoid the overlapping of multipath signals it has considered:

- The emitter and the hydrophone were fixed and aligned using a steel cable in a diagonal line at 50 cm depth, as presented in Figure 6, to reduce the probability of receiving reflections, assuming that the hydrophone is directional.
- The signal sent was a sine wave with 20 cycles of 250, 500, 750 and 1000 kHz with enough interval between bursts (10 ms) to avoid that the received echoes overlap with each other.

Figure 6 shows the experimental setup scheme with the hydrophone and the transducer positions for the TVR and the Spread Angle Response tests.

For the drive signal, it was used as a Signal Generator B&K Precision 4053 amplified by a 5 W Class B Push-Pull symmetric voltage amplifier with a maximum gain of 12 dB. The hydrophone was a Cetacean ResearchTM C304XR, with a transducer sensibility of −181 dB, re 1 V/µPa, a linear frequency range (±3 dB) of 0.012–1000 kHz and usable frequency range (+3/−12 dB) of 0.005–2000 kHz with a 2nd order active band-pass filter from 1 to 2000 kHz with 6 dB gain. A digital oscilloscope PicoScope 4227, 100 MHz, was used to record the measurements.

**Figure 7:**
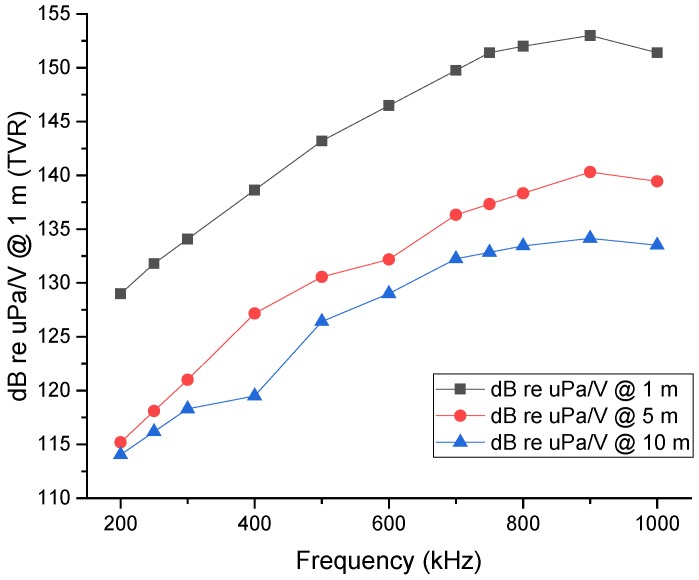
Transmitted voltage response as function of frequency for 1, 5, and 10 m distance between 200 and 1000 kHz.

Figure 7 shows the transducer response, between 200 kHz and 1 MHz, for 1, 5 and 10 m of distance. The PVDF transducer results were calibrated through the hydrophone sensibility.

The Transmission Voltage Response (TVR) at 1 m distance starts at 130 dB for 200 kHz and increases to 150 dB for 750 kHz and then displays an almost flat response up to 1 MHz. For a distance of 5 and 10 m, the response is similar however with a normal constant attenuation due to distance attenuation. At 200 kHz both responses have around 115 dB and then start to rise, achieving 130 dB at 5 m and 135dB at 10 m. Nevertheless, all tests show an almost flat response between 750 kHz and 1 MHz. 

**Figure 8:**
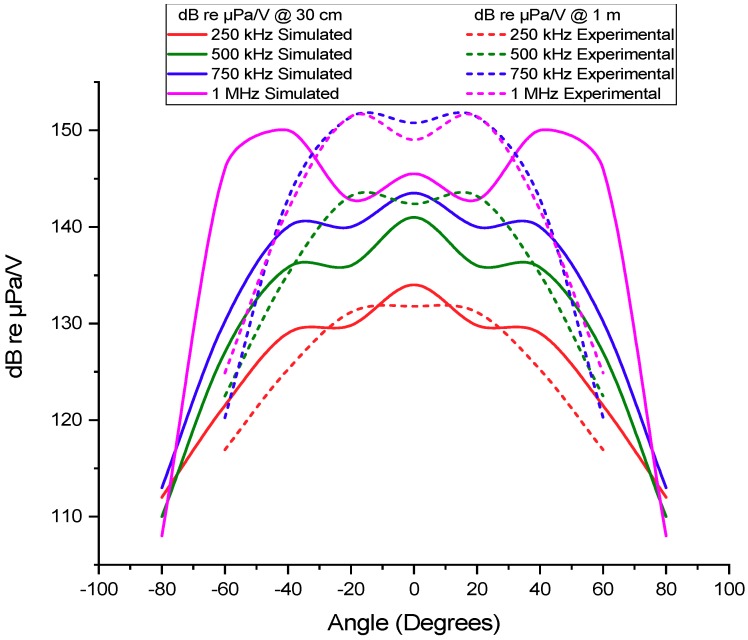
Experimental and simulated results of the unnormalized radiation diagram for 250 kHz, 500 kHz, 750 kHz, and 1 MHz frequencies. The dashed line corresponds to the experimental results (dB re µPa/V@1 m) and the continues line to the simulation results (dB re µPa/V@30 cm).

Figure 8 shows the measured TVR at 1 m as a function of the beam spreading angle for 250, 500, 750 and 1000 kHz obtained in the experimental tests and for comparison the results from the FEM simulations.

Before comparing the simulation results with the experimental ones, it is important to remind that there is some discrepancy in the between simulation and the experimental conditions since the simulation was performed with 30 cm, while the tests were performed at 1 m and in the experiment exists the frequency-dependent amplification and Transmission Loss. 

In terms of angle response, the beam width was considered up to 6 dB above the maximum TVR. The experimental results show that the transducer has a beam wider than the expected 70°, but not as wide as the one obtained in the simulations, which present a spread angle of 110°. In terms of bandwidth, a quality factor of 1.8 centered in 755 kHz is presented at 1m, demonstrating high bandwidth properties. Through the simulation, it was possible to predict the transducer beam angle for all frequencies and also the increase of pressure levels in the lateral lobes (at 20°) for frequencies above 1 MHz (inclusive). The results obtained were as expected and demonstrate a high potential for applications in short-range broadband underwater communications since the transducer presents a high bandwidth and beam-width.

## 6. Field Test Experimental Setup and Results

**Figure 9:**
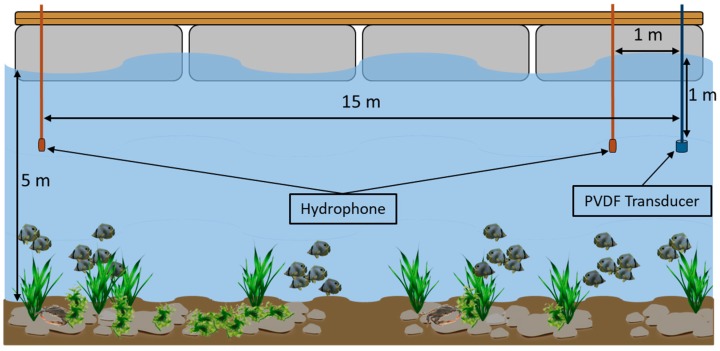
Field Test Experimental setup diagram carried out in floating platform in Ria de Formosa, Algarve with 5 m deep shallow water channel. The emitter transducer was fixed at 1 m deep and the hydrophone was placed at 1 and 15 m distance with 1 m deep.

Field tests allow transducer evaluation for communications purposes, to verify the usability of the bandwidth and analyze the behavior, such as operational distance and beam width in real conditions. The field tests were carried out in Ria de Formosa, Algarve, Portugal (37°00′11.2″ N 7°59′09.6″ W). A floating platform in a 5 m deep shallow water channel was used. The emitter transducer was fixed at 1 m depth and the hydrophones were placed at two different distances, 1 and 15 m, and 1 m depth. In the field tests, it was not necessary to ensure perfect alignment between the emitter and the receiver, since one of the test objectives was to verify if the emitter beam width allowed to maintain the communication continuity with small transducer movement due to surface waves. Figure 9 shows the test setup.

The same calibrated hydrophone was used as in the pool test, a Cetacean Research^TM^ C304XR hydrophone, with a transducer sensibility of −181 dB, re 1 V/µPa and a linear Frequency Range (±3 dB) of 0.012–1000 kHz. The filter consists of a 2nd order active band-pass from 1 to 2000 kHz with 6 dB gain, mainly to reduce typical interference above 1 kHz but also high-frequency noise. Two Red Pitaya boards were used for signal acquisition and signal generation. The Red Pitaya ADC and DAC had a maximum sample rate of 125 MSPS and it was set a 64 prescaler, resulting in effective sample rate of 1953125 SPS. Drive signals, from the Red Pitaya DAC, are amplified by a 5 W Class-B Push-Pull symmetric voltage amplifier with ±10V of output signal. The signal processing was performed by MATLAB in a PC, which was connected to the Red Pitaya through an Ethernet cable.

**Figure 10:**
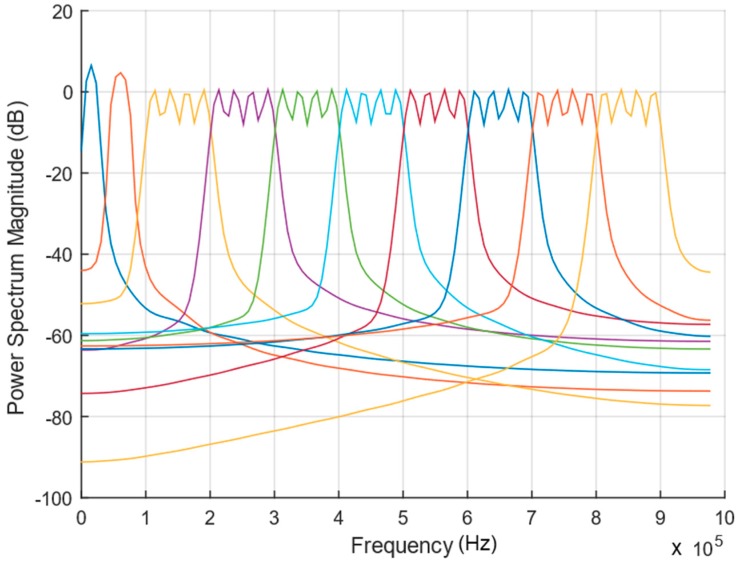
Power spectrum of the 10 chirp signals selected for the field tests.

To evaluate the transducer performance in the field and behavior of the available bandwidth, 10 chirp signals (from f_1_ to f_2_); 10→20, 50→60, 100→200, 200→300, 300→400, 400→500, 500→600, 600→700, 700→800 and 800→900 kHz were selected, with power spectrum as represented in Figure 10. Chirp signals were used, to avoid possible interference that could arise from surrounding noise when using single-frequency signals.

Each chirp signal was transmitted in burst mode, with a signal duration of 512 µs and a repetition period of 1024 µs, during a total time of 8 ms (8 equal chirp bursts were sent). 

**Figure 11:**
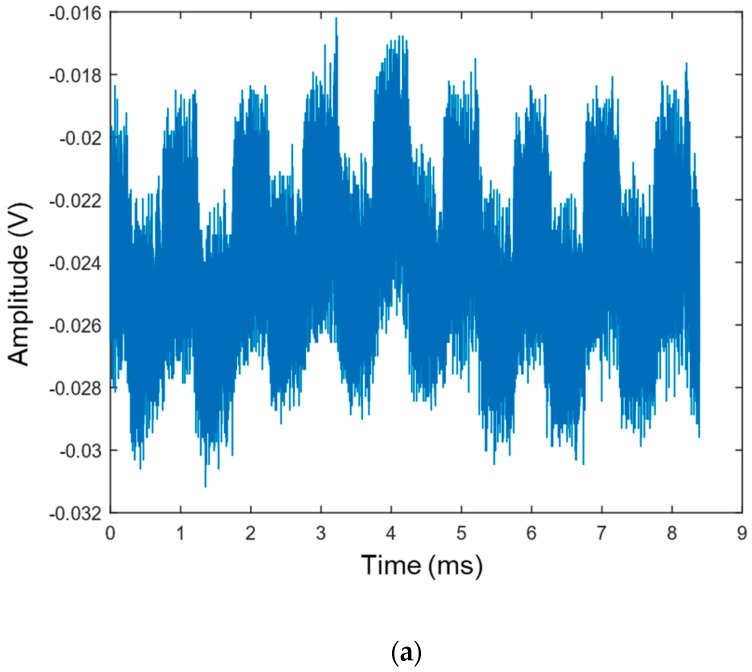

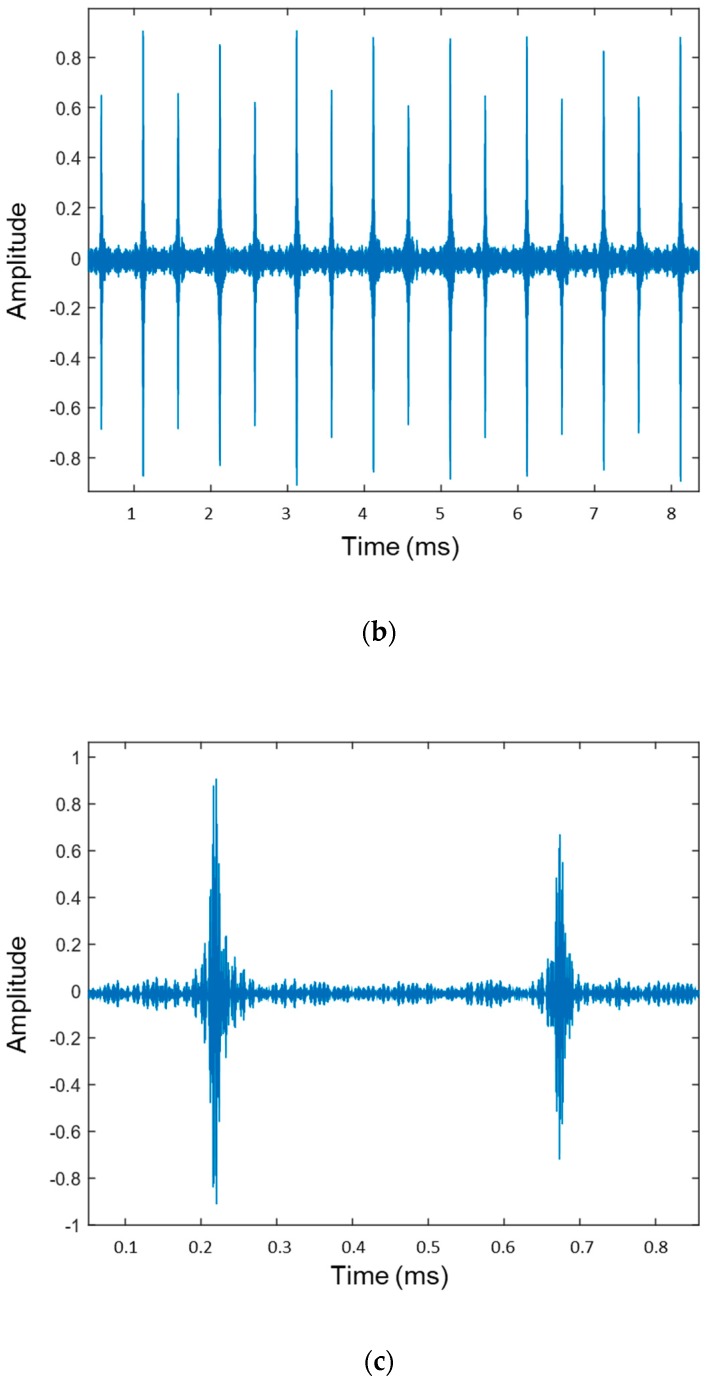
Received signal on the 15 m test: (**a**) Received signal for the 800→900 kHz chirp; (**b**) Cross-correlation result from signal received; (**c**) Zoom of the Cross-correlation result.

On the receiver side, the signal was cross-correlated using MATLAB, with 10 selected chirp signals samples to detect which chirp was received. The test was carried out 3 times, for 1 and for 15 m, for each of the 10 selected chirp frequency ranges (resulting in a total of 60 tests), and each test was registered. Figure 11a displays one of the 800→900 kHz chirp received signals on the 15 m test.

Figure 11b shows cross-correlation results between an 800→900 kHz chirp signal sample and received signal of Figure 11a,c, shows a zoom from Figure 11b. In Figure 11b, there are 16 correlation peaks visible, instead of the expected 8, meaning that 16 signals were received, yet only 8 were sent. This could be justified by the occurrence of echoes. Such echoes are delayed for approximately 450 µs later and could only at the buoys placed at the lateral borders of the peer since the transducer has a vertical beam spread angle of 10° and in the horizontal plane was more than 70°.

**Figure 12:**
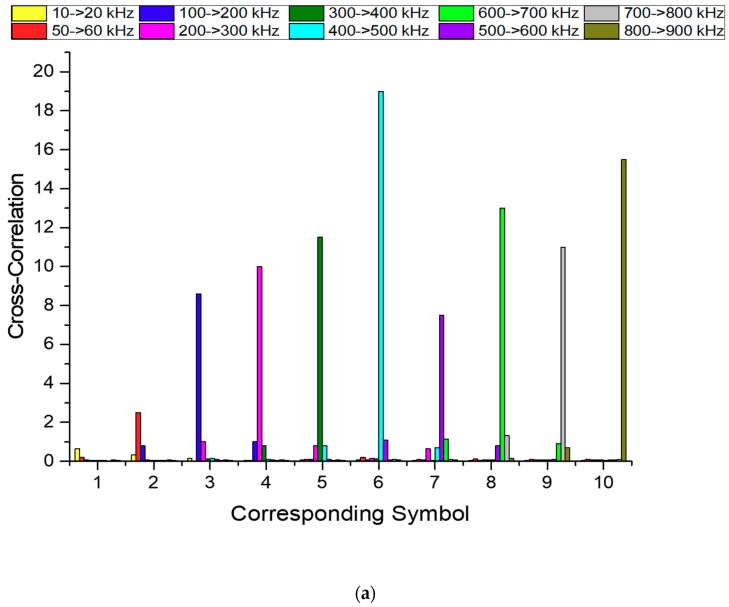

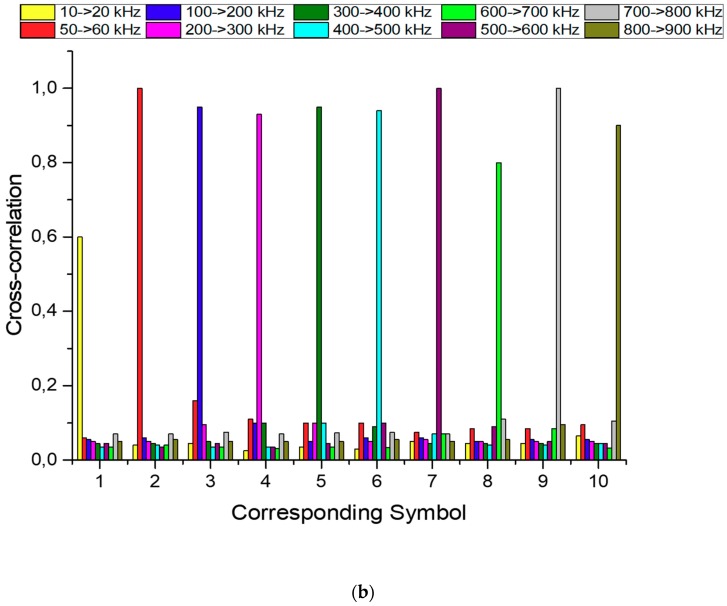
Cross-correlation result for the 10 chirp signals transmitted with the 10 chirp samples at: (**a**) 1 m distance and (**b**) 15 m distance.

The cross-correlation tests allowed to verify the bandwidth usability from 10 to 900 kHz. To simplify the evaluation the results were compiled in Figure 12, where each chirp signal received is associated to a corresponding symbol, defined as follows: 1 to 10→20, 2 to 50→60, 3 to 100→200, 4 to 200→300, 5 to 300→400, 6 to 400→500, 7 to 500→600, 8 to 600→700, 9 to 700→800 and 10 to 800→900 kHz. Each received chirp signal was cross-correlated with the 10 chirp samples, which means that, for example, the red bars correspond to the cross-correlation result between the 50→60 kHz samples with the 10 chirps received. Therefore, in each 1 to 10 symbol the highest bar corresponds to the transmitted chirp. The results are presented in Figure 12a,b respectively.

From the results shown in Figure 12a, it is possible to conclude that all signals were successfully identified, even though, the transducer demonstrates a low performance at frequencies below 100 kHz, which is reflected in the low cross-correlation amplitude for the 10 to 20 kHz chirp signal. The abnormal decrease of power above the 6-symbol is possibly due to the geometric instability caused by the water dynamics and the pear motion, which causes small changes in the geometric setup. Those small changes of geometry have a strong impact on the received signal when the emitter and receiver are close, but lose significance at larger distances. Considering a signal with 1 MHz at 1 m, the isonified vertical area have only 8.6 cm height from de center, while at 15 m have 130 cm. Moreover, the instability affects more the high frequencies, since the transducer divergence angle reduces with the frequency.

In Figure 12b the results of the 10 to 20 kHz chirp signal maintain their respective amplitude in contrast to the remaining frequencies that suffered a drastic amplitude reduction in the results at 15 m, this is due to the fact that the low frequencies are less affected in terms of attenuation over distance.

In Figure 12b, all signals were successfully identified using cross-correlation. At 15 m, all results display similar amplitude values, and this is because the transducer has a better response at high frequencies while the medium exhibits exponential attenuation with increasing frequency.

## 7. Conclusions

The present work describes the development of a high frequency, wide beam and wideband PVDF acoustic transducer and its characterization as emitter. After the statement of operating principles, development and design it was possible to predict and optimize the transducer performance through simulation using a finite element method. Through acoustic and electric characterization, it was possible to demonstrate transducer performance in terms of beam angle and operational frequencies. The transducer shows a beam divergence angle above 70° (horizontally) and a usable frequency band between 100 kHz and 1.5 MHz with a maximum TVR of 150 dB re µPa/V. Finally, the transducer was tested in a real scenario at sea, carried out in the Ria de Faro, Algarve, Portugal. 10 chirp signals between 10 and 900 kHz at 1 and 15 m distance, were tested. The received signals were cross-correlated with emitted signals and the results show that all symbols were successfully identified. Therefore, the transducer presents a linear bandwidth of 250 kHz and usable bandwidth of 1 MHz. It can transmit up to 15 m with low power consumption and it is possible to be used in array and to implement an omnidirectional emitter. The results support the fact that the developed transducer is suitable for underwater broadband wireless communications. In future works, this transducer will be used to transmit high-quality real-time video.

## 8. Patents

The work reported in this manuscript results from an international patent application with the No. PCT/IB2018/054471.

## Figures and Tables

**Table 1:** Comparison of some characteristics of PZT-5H and polymer ultrasound transducer (PVDF) [4].

Physical Property	PZT-5H	PVDF
Sound Speed (m/s)	4.2 × 10^3^	2.25 × 10^3^
Density (kg/m^3^)	7.5 × 10^3^	1.47 × 10^3^
Acoustic Impedance Z (10^6^ kg/m^2^s)	31.5	3.3075
Relative Dielectric constant εr	3100	12
Piezoelectric Coefficient *d*_33_ (C/N)	5.12 × 10^−10^	3.40 × 10^−11^
Elastic compliance coefficient $S_{33}^{E}$ (1/Pa)	2.07 × 10^−11^	4.72 × 10^−10^
Reflected wave (%)	88%	28.7%

**Table 2:** Transducer requirements [12].

Characteristics	Value
Frequency	Up to 1 MHz
Cylinder radius	7.5 cm
XY plane beam spread	70°
XZ plane beam spread	10°

**Table 3:** Parameters configuration for the acoustic transducer finite element simulation.

Parameter	Value
Propagation medium	Freshwater, 20 °C of temperature.
Active element	PVDF film
Thickness	200 µm
Internal mechanic boundary	Free movement setup
Internal electric boundary	Connected to the GND
External mechanic boundary	Fixed to a hard surface
External electric boundary	1 V sine wave signal
Wave signal frequency	250 kHz, 500 kHz, 750 kHz and 1 MHz
