# Erratum: Martins, M.S., et al. Wideband and Wide Beam Polyvinylidene Difluoride (PVDF) Acoustic Transducer for Broadband Underwater. *Sensors* 2019, *19*, 3991

**DOI:** 10.3390/s19224928

**Published:** 2019-11-12

**Authors:** Marcos S. Martins, Carlos L. Faria, Tiago Matos, Luís M. Goncalves, José Cabral, António Silva, Sérgio M. Jesus

**Affiliations:** 1 MEMS-UMinho, University of Minho, Campus of Azurém, 4800-058 Guimarães, Portugal; carlosfaria@dei.uminho.pt (C.L.F.); b7567@dei.uminho.pt (T.M.); lgoncalves@dei.uminho.pt (L.M.G.); cabral@dei.uminho.pt (J.C.); 2 LARSyS, University of Algarve Campus de Gambelas, 8005-139 Faro, Portugal; asilva@ualg.pt (A.S.); sjesus@ualg.pt (S.M.J.)

The authors wish to make the following erratum to this paper [1]:

Equations (1), (7), and (9) are incorrect and must be replaced by the following equations:

(1) $$R=\;\frac{Z_{2}-Z_{1}}{Z_{2}+Z_{1}}$$

(7) p=2πcρfξ

(9) $$nt_{p}\leq \frac{1}{2\pi c\rho S_{33}^{E}f}$$

The authors apologize for this literal mistake, but emphasize that the content of the article is still correct, since all calculations were performed with the correct equations. The manuscript will be updated and the original will remain online on the article webpage, with a reference to this Erratum.
